# Upregulation of serum circular RNA FUNDC1 and TNF-α in Behçet’s disease: potential diagnostic biomarkers

**DOI:** 10.1038/s41598-026-66489-y

**Published:** 2026-08-22

**Authors:** Rehab Elsayed Marzouk, Marwa Kamel, Olfat G. Shaker, Mohammed Ali Gameil, Yasmine M. Amrousy, Mai A. El Kosaier, Reem Abdelrahman, Noha O. Shawky, Laila Mahdi

**Affiliations:** 1 Department of Medical Biochemistry and Molecular Biology, Faculty of Medicine, Capital University (Formerly Helwan University), Cairo, Egypt; 2 Department of Medical Biochemistry and Molecular Biology, Faculty of Medicine, Cairo University, Cairo, Egypt; 3 Department of Internal Medicine and Endocrinology, Faculty of Medicine, Mansoura University, Mansoura, Egypt; 4 Department of Clinical and Chemical Pathology, Faculty of Medicine, Capital University (Formerly Helwan University), Cairo, Egypt; 5 Department of Rheumatology, Rehabilitation and Physical Medicine, Faculty of Medicine, Mansoura University, Mansoura, Egypt; 6 Department of Microbiology and Immunology, Faculty of Medicine, Capital University (Formerly Helwan University), Cairo, Egypt; 7 Department of Medical Physiology, Faculty of Medicine, Capital University (Formerly Helwan University), Cairo, Egypt

**Keywords:** CircRNA-FUNDC1, Tumor necrosis factor-alpha, Behçet’s disease, BDCAF, Biomarkers, Diseases, Immunology, Medical research

## Abstract

Behçet’s disease (BD) is an inflammatory autoimmune disease characterized by relapsing genital ulcers, ocular involvement, and intestinal disorders. Evaluate serum levels of circular RNA (circRNA) FUNDC1 and tumor necrosis factor-alpha (TNF-α) in BD patients and compare them accordingly to healthy individuals. One hundred participants were enrolled in this study and subdivided equally into BD patients and healthy matched individuals. A five mL sample of blood was drawn from each subject and analyzed to measure circRNA-FUNDC1 and TNF-α. Full clinical investigations have been performed, and the Behcet Disease Current-Activity Form score (BDCAF) has been calculated. circRNA-FUNDC1 and TNF-α were measured by quantitative real time PCR and enzyme-linked immunosorbent assay (ELISA) respectively. An up-regulation of circRNA-FUNDC1 as well as TNF-α (*p* < 0.0001) in BD group were reported compared to controls. Both biomarkers tended to elevate with the activity of the disease as levels were higher in active BD patients than inactive patients. The level of circRNA-FUNDC1 (FC) tended to be higher in patients with negative musculoskeletal and central nervous system (CNS) manifestations than those with positive manifestations (p-values = 0.007, 0.037, respectively). The level of TNF-α was reported higher in those with oral ulcer with (*p* = 0.041), also in patients with positive skin manifestations than in negative patients (*p* = 0.025). The level of circRNA-FUNDC1 (FC) was higher in patients who took Azathioprine and steroids (*p* = 0.019, 0.035) and lower in patients who took cyclosporin (*p* = 0.049). TNF-α tended to elevate in patients who took Hydroxychloroquine than those who did not (*p* = 0.040). The upregulated levels of circRNA-FUNDC1 and TNF-α may have a potential role in improving our understanding of the molecular mechanisms underlying BD.

## Introduction

Behçet’s disease (BD) is a rare, multisystem chronic disorder with no specific histological or laboratory markers for diagnosis. Instead, clinical criteria are used to establish the diagnosis, recently updated by an international study group^1–3^.

Circular-RNAs (circRNAs) as type of non-coding RNAs, play significant roles in the regulation of gene expression, modulate immune responses, and control inflammation^4,5^. Circular RNA FUNDC1 (circRNA-FUNDC1) is derived from the FUN14 domain-containing 1 (FUNDC1) gene, which is involved in mitophagy and cellular stress responses. As a circular RNA, it forms a stable, covalently closed structure, making it resistant to degradation^6,7^.

FUNDC1 is an intracellular mitochondrial outer membrane protein expressed in immune and vascular-relevant cells, including macrophages, T lymphocytes, and endothelial cells, where it regulates mitophagy under conditions of hypoxia and inflammatory stimulation^8,9^.

Under oxidative stress and inflammation, FUNDC1-dependent mitophagy reduces mitochondrial damage and reactive oxygen species (ROS) accumulation, thereby modulating downstream inflammatory signaling pathways that contribute to cytokine production and vascular inflammation relevant to Behçet’s disease^8,10,11^.

CircRNAs can act as sponges for microRNAs, influencing the levels of miRNAs that regulate pro-inflammatory signaling molecules like and interleukin 6 and tumor necrosis factor alpha^4,5^.

Tumor necrosis factor-alpha (TNF-α) is an essential pro-inflammatory cytokine that contributes to immune regulation and inflammation. It is produced by T-cells and macrophages in response to infections and autoimmune triggers, driving tissue damage and immune activation. Cytokines such as TNF-α, showed a crucial function in the development of autoimmune diseases as well as the therapeutic potential of cytokine-targeted therapies. In addition, TNF inhibitors are widely used to manage these conditions by reducing inflammation and improving symptoms^12–14^.

Currently, to the best of our knowledge, there are no direct studies or evidence linking circRNA-FUNDC1 in combination with TNF-α together specifically to Behçet’s disease. However, circRNA-FUNDC1 can regulate inflammation by serving as a competitive endogenous RNA (ceRNA), binding to microRNAs in a sponge-like manner that modulate proinflammatory signaling pathways^6,7^.

The core purpose of this study is to evaluate the levels of circRNA-FUNDC1 and TNF-α in the serum of BD patients and compare them accordingly to healthy individuals, aiming to analyze the potential role of these serum markers in BD.

## Subjects and methods

Fifty participants meeting the criteria outlined in 1975 Declaration of Helsinki were included in this study, with an allowable absolute error/precision margin of 1.37% and a confidence level (CI) of 95% with total sample size is 49.23. Agreeing on a total of 100 subjects subdivided equally into two groups with matched age and sex, patients with Behçet’s disease and controls to be in the study. The study approval was obtained from the Faculty of Medicine, Mansoura University, with approval code R.24.08.2752 on 23/10/2024. A written informed consent was acquired from every participant. All methods were performed in accordance with the relevant guidelines and regulations.

Participants were enrolled from both in-and-outpatient clinics of the Internal Medicine Department, Rheumatology & Rehabilitation, and Physical Medicine Department, Mansoura University Hospitals. The study was conducted at Department of Medical Biochemistry and Molecular Biology, Faculty of Medicine, Kasr Alainy Hostipals.

The Behcet’s Disease Current Activity Form (BDCAF) was used for assessment of behcet disease activity by examining the history and clinical symptoms caused by BD in the last four weeks using the following 12 components: headache, oral aphthous, genital ulcers, skin erythema, skin pustules, arthralgia, arthritis, nausea/vomiting/abdominal pain, diarrhea with altered/frank blood per rectum, eyes, central nervous system, and major blood vessels. Each component is given a score of absent (0) or present^1^. The BD activity index (BDCAF) is calculated by adding the scores of each component, with a maximum score of 12. A total score ≥ 2 is defined as active BD, whereas < 2 is defined as inactive BD^15^.

The inclusion criteria compromised female and male adult patients with a confirmed diagnoses of Behçet’s disease based on the international criteria for Behçet’s disease (ICBD), defined by the International Team for the Revision of ICBD^16^.

Patients with any other inflammatory disease or chronic infectious disorder were excluded in the study. Pregnant and lactating females, cognitively impaired, or mentally disabled subjects were also excluded from this study.

For the healthy control group, demographic data (age and sex) and selected clinical information, including family history and associated diseases/comorbidities, were recorded. Other clinical variables were not systematically collected, as the controls served as a reference group for biomarker comparison.

### Molecular biology techniques for serum biomarkers

For each participants, a five mL blood sample was collected and centrifuged for serum separation to detect the expression and concentration levels of circRNA-FUNDC1 and TNF-α by quantitative PCR and ELISA (enzyme linked immunosorbent assay) respectively.

### Expression level of circRNA-FUNDC1 by real-time qPCR (quantitative polymerase chain reaction)

By using Qiagen, total RNA was extracted from separated serum. Linear RNAs have been degraded using an exonuclease such as ribonuclease R (RNase R). RNase R from E. coli, is a 3′to 5’ exoribonuclease that can digest linear RNA. One µg of extracted RNA was incubated with 3 U of RNase R (LGC, Cat# RNR07250) (Sigma Aldrich, Germany**)** in 1X RNase R reaction buffer at 37 °C for 20 min, then heat inactivation at 70 °C for 10 min. Samples underwent RNA quantitation and purity evaluation using the ND-1000 NanoDrop (spectrophotometer (NanoDrop Technologies, Inc., Wilmington, USA). Afterward, a reverse transcription (RT) on total RNA was done in a final volume of 20uL using the miScript II RT kit (Qiagen, Valencia, CA, USA). For the detection of the expression level of circRNA-FUNDC1, quantitative real-time PCR (qRT-PCR) took place. Then, SYBER Green PCR kit was ued to detect the relative quantitative by using Qiagen, catalog 218073).

The sequence of the primers for circRNA-FUNDC1 (human circRNA-FUNDC1 forward primer: 5′-CCAGAGTCTCTAGGGCAAGG-3′, reverse primer: 5′ - TGCTTTGTTCGCTCGTTTCT-3′) were used for quantitative real time PCR. GAPDH (GAPDH forward, 5′-TGCACC ACC AAC TGC TTA GC-3′, reverse: 5′-GGC ATG GAC TGT GGT CAT GAG-3′ (Ref Seq: NM_001256799.3) were used as reference gene. Ct of GAPDH was subtracted from the values of Ct of circRNA-FUNDC1 for all participants. Then Ct values was performed by subtracting the control Ct values from the patients’ values. The relative quantitation or fold changes (FC) were calculated for the circular RNA using the method of 2^−ΔΔCt^. After the completion of qRT-PCR cycles, melting curves were analyzed to confirm the targeted circRNA-FUNDC1 expression.

### Detection of the concentration level of TNF-α using ELISA

For measuring the concentration level of TNF-α in serum, enzyme-linked immunosorbent assay (ELISA) was used (BT LAB ELISA, Catalog no. E0082Hu).

### Statistical analysis of data

On windows 8.1 and by using Statistical Package of Social Science (SPSS) software version 22.0 data was analyzed. Kruskal-Wallis test for comparing groups having quantitative variables was used. Mean average, standard deviation (SD), and range were used for quantitative data. Frequency distribution and percentages were used for qualitative data. Independent Student t-test was used for the analysis of parametric data, while for nonparametric data Chi-square test (χ2) was used. Multiple pairwise comparisons were performed where the Benjamini-Hochberg false discovery rate (FDR) procedure was applied to control for Type I error. Pearson correlation test was used for correlating quantitative parameters at a significance of ≤0.05. To further evaluate potential treatment-related confounding while minimizing multicollinearity, additional multivariate models included only medications that demonstrated significant associations in the univariate analyses by which regression coefficients (β), 95% confidence intervals (CI) and coefficients of determination (R²) were reported.

## Results

### Demographic and clinical characteristics of the studied populations

Afifty behçet patients 6(12%) females and 44(88%) males with a mean of age 33.44 ± 7.8 years; another 50 controls as 40(80%) males and 10(20%) females with a mean of age 33.86 ± 4.0 were included in the present study. No significant difference was reported in neither age nor sex between groups with *p* > 0.05.

Symptoms were diagnosed and recorded for all patients including oral and/or genital ulcer and organ involvement (skin, musculoskeletal, gastrointestinal tract (GIT), ocular, central nervous system (CNS), and vascular manifestations) (Table 1).

**Table 1:** Clinical characteristics of Behçet patient group.

Variable	Behçet patients (*N* = 50)
Clinical symptoms	
Oral ulcer	23 (46%)
Genital ulcer	6 (12%)
Organ involvement	
Skin manifestations	15 (30%)
Musculoskeletal manifestations	30 (60%)
GIT manifestations	11 (22%)
Ocular manifestations	32 (64%)
CNS manifestations	16 (32%)
Vascular manifestations	6 (12%)
Immunosuppressants	
Azathioprine	20 (40%)
Cyclosporin	20 (40%)
Hydroxychloroquine	4 (8%)
Drugs	
Steroids	39 (78%)
Anti TNF	10 (20%)
Medication patterns	
Colchicine	38 (76%)
BDCAF Patient’s Index Score: 3.08 ± 1.85	
Score (Inactive: Active Behcet Disease)	0.80 ± 0.42:3.64 ± 1.61 (*p* = 0.004*)

Data shown as N (%), GIT: Gastrointestinal Tract, CNS: Central nervous system, Anti TNF: anti-tumor necrosis factor, BDCAF: Behcet Disease Current Activity Form score. BDCAF Patient’s Index Score is shown as mean ± SD. Independent Student T-Test is used. BDCAF Patient’s Index Score≥2 active Behcet Disease, score < 2 inactive Behcet Disease.

In addition, treatment and medication patterns have been recorded. Patients undertook immunosuppressants such as Azathioprine, Cyclosporin, and Hydroxychloroquine, covering 20 (40%), 20(40%), and 4(8%), respectively. While 39(78%) of the patients were under the treatment of Steroids, only 10 (20%) were using anti-tumor necrosis factor (Anti-TNF). For other medication patterns, 38 (76%) of the patients were using Colchicine (Table 1).

Patient’s Index Score BDCAF, had been evaluated where all BD patients had a mean and standard deviation of 3.08 ± 1.85. When patients were subclassified into inactive and active Behçet disease according to a significant difference as regards activity of the disease, with *p* = 0.004 (Table 1).

### CircRNA-FUNDC1 and TNF-α serum biomarkers levels for Behçet disease and healthy control groups

**Fig. 1:**
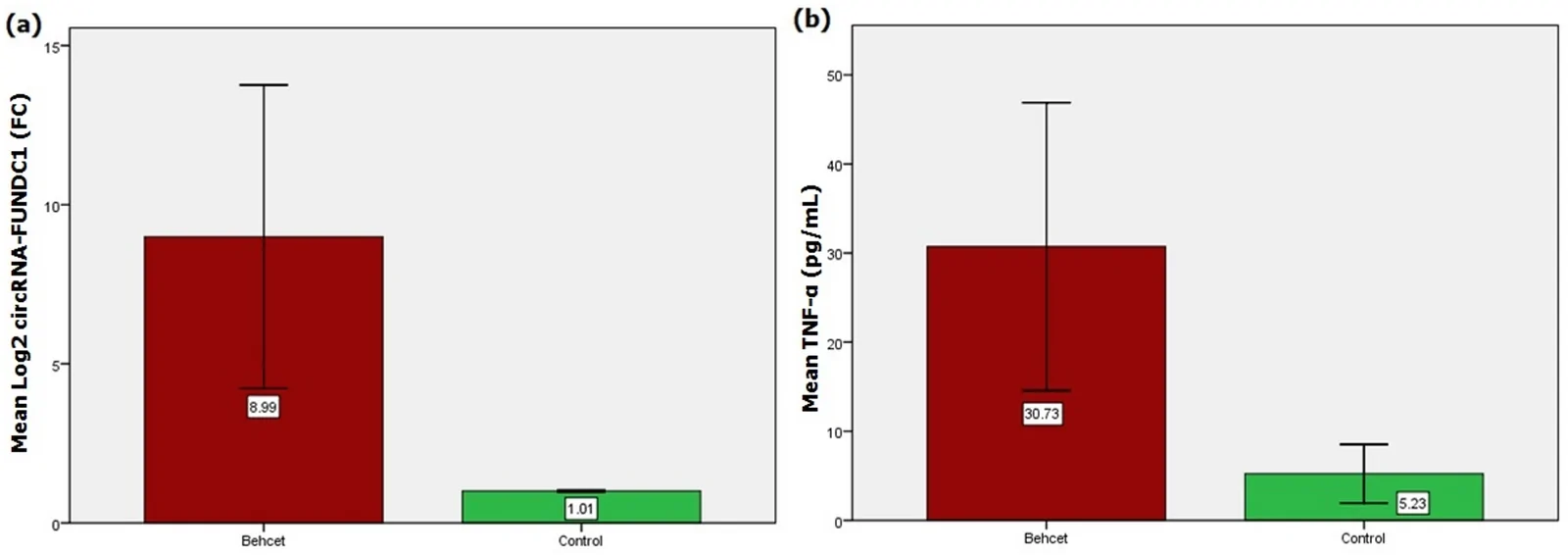
Serum levels of the study biomarkers between Behçet patients and healthy controls (**a**) expression level of log2 circRNA-FUNDC1 (FC), (**b**) serum concentration level of TNF-α.

CircRNA-FUNDC1 and TNF-α were assessed and comparatively analyzed` between BD patients and controls. Statistically significant variations were identified between the two groups with respect to both biomarkers, where log2 CircRNA-FUNDC1 and TNF-α were higher in patients group compared to controls (*p* < 0.0001) for both biomarkers (Table 2; Fig. 1).

**Table 2:** CircRNA-FUNDC1(FC) and TNF-α (pg/mL) serum biomarkers levels for Behçet and healthy control groups.

Serum biomarkers	Behçet patients (*N* = 50)	Healthy control (*n* = 50)	*p*-value
Log2 circRNA-FUNDC1(FC)	8.99 ± 4.76	1.01 ± 0.03	< 0.0001*
TNF-α (pg/mL)	30.73 ± 16.14	5.23 ± 3.2	< 0.0001*

Data shown as mean ± SD, Independent sample T-tests used, * significant (*P*≤0.05).

No significant difference was reported between the expression level of circRNA-FUNDC1 in serum and clinical data among the BD patients, including (gender, clinical symptoms, either oral or genital ulcer) where *p* > 0.05. Even no significant difference was detected as regards organ involvement (skin, GIT, ocular, or vascular), where p-value > 0.05. However, a significant difference was detected between log2 circRNA-FUNDC1 in patients with negative musculoskeletal and CNS manifestations, with higher expression levels of log2 circRNA-FUNDC1 than in positive patients with manifestations respectively *p* = 0.007 and 0.0037 (Table 3).

**Table 3:** Expression level of Log2 circRNA-FUNDC1(FC) and concentration level of TNF-α for Behçet patients with demographic and clinical characteristics.

Variable		Log2 circRNA-FUNDC1(FC)	*p*-value	TNF-α (pg/mL)	*p*-value
Sex	Female (*N* = 6)	8.77 ± 2.32	0.087	40.75 ± 18.69	0.49
	Male (*N* = 40)	9.02 ± 5.02		29.35 ± 15.51	
Clinical symptoms					
Oral ulcer	Negative (*N* = 27)	9.73 ± 4.12	0.12	28.06 ± 13.78	**0.041** ^*^
	Positive (*N* = 23)	8.12 ± 5.39		33.85 ± 18.36	
Genital ulcer	Negative (*N* = 44)	9.48 ± 4.63	0.87	30.89 ± 15.74	0.34
	Positive (*N* = 6)	5.37 ± 4.46		29.51 ± 20.53	
Organ involvement					
Skin manifestations	Negative (*N* = 35)	8.52 ± 4.50	0.47	27.41 ± 14.50	**0.025** ^*^
	Positive (*N* = 15)	10.08 ± 5.33		38.46 ± 17.60	
Musculoskeletal manifestations	Negative (*N* = 20)	10.0 ± 6.23	**0.007** ^*^	31.38 ± 13.87	0.24
	Positive (*N* = 30)	8.32 ± 3.43		30.28 ± 17.72	
GIT manifestations	Negative (*N* = 39)	9.14 ± 5.21	0.19	30.52 ± 15.33	0.27
	Positive (*N* = 11)	8.47 ± 2.81		31.43 ± 19.58	
Ocular manifestations	Negative (*N* = 18)	9.07 ± 3.97	0.66	31.34 ± 16.40	0.84
	Positive (*N* = 32)	8.95 ± 5.22		30.38 ± 16.25	
CNS manifestations	Negative (*N* = 34)	9.13 ± 5.54	**0.037** ^*^	27.22 ± 15.75	0.88
	Positive (*N* = 16)	8.70 ± 2.53		38.17 ± 14.80	
Vascular manifestations	Negative (*N* = 44)	9.06 ± 5.0	0.28	30.60 ± 16.45	0.45
	Positive (*N* = 6)	8.47 ± 2.69		31.58 ± 15.01	
Immunosuppressants					
Azathioprine	No (*N* = 30)	7.47 ± 3.21	**0.019** ^*^	33.73 ± 15.80	0.72
	Yes (*N* = 20)	11.26 ± 5.81		26.21 ± 15.99	
Cyclosporin	No (*N* = 30)	9.74 ± 5.62	**0.049** ^*^	27.50 ± 16.11	0.08
	Yes (*N* = 20)	7.87 ± 2.84		35.55 ± 15.34	
Hydroxychloroquine	No (*N* = 46)	9.08 ± 4.93	0.28	29.34 ± 15.03	**0.040** ^*^
	Yes (*N* = 4)	8.0 ± 2.29		46.55 ± 22.54	
Drugs					
Steroids	No (*N* = 11)	6.82 ± 3.16	**0.035** ^*^	28.84 ± 14.61	0.38
	Yes (*N* = 39	9.60 ± 4.99		31.25 ± 16.69	
Anti TNF	No (*N* = 40)	9.57 ± 4.83	0.063	30.88 ± 16.17	0.89
	Yes (*N* = 10)	6.68 ± 3.89		30.09 ± 16.88	
Medication patterns					
Colchicine	No (*N* = 12)	8.16 ± 5.51	0.86	28.59 ± 13.96	0.22
	Yes (*N* = 38)	9.25 ± 4.55		31.39 ± 16.89	

Data shown as mean ± SD, Independent sample T-tests used, * significant (*P*≤0.05). Due to the large number of comparisons performed, p-values were additionally assessed using the Benjamini-Hochberg false discovery rate (FDR) procedure. None of the associations remained statistically significant after adjustment (all q-values > 0.05); therefore, these findings should be considered exploratory and hypothesis-generating.

In addition, the level of log2 circRNA-FUNDC1 was reported higher in BD patients who received Azathioprine as an immunosuppressant than in patients who did not, with *p* = 0.019. While patients who did not take Cyclosporin had higher levels of log2 circRNA-FUNDC1 than those who took Cyclosporin (*p* = 0.049). In addition, there was a significantly higher level of log2 circRNA-FUNDC1 in patients who received Steroids than in those who did not, with p-value = 0.0035 (Table 3).

The concentration level of TNF-α was higher in patients having oral ulcers than those without ulcers, with a significance of *p* = 0.041. As for organ manifestations, there was only a significant difference as regards patients with skin manifestations involvement than those who had negative skin issues, with *p* = 0.025.

In the present study, TNF-α concentration levels were higher in patients receiving Hydroxychloroquine as an immunosuppressant, with *p* = 0.040 (Table 3).

After applying the FDR correction for multiple comparisons, the previously observed associations between circRNA-FUNDC1 and the associated clinical variables, as well as between TNF-α expression levels and the associated clinical variables, were no longer remained statistically significant after adjustment (all q-values > 0.05); therefore, these findings should be considered exploratory and hypothesis-generating.

### Association of circRNA-FUNDC1 and TNF-α with disease activity

**Fig. 2:**
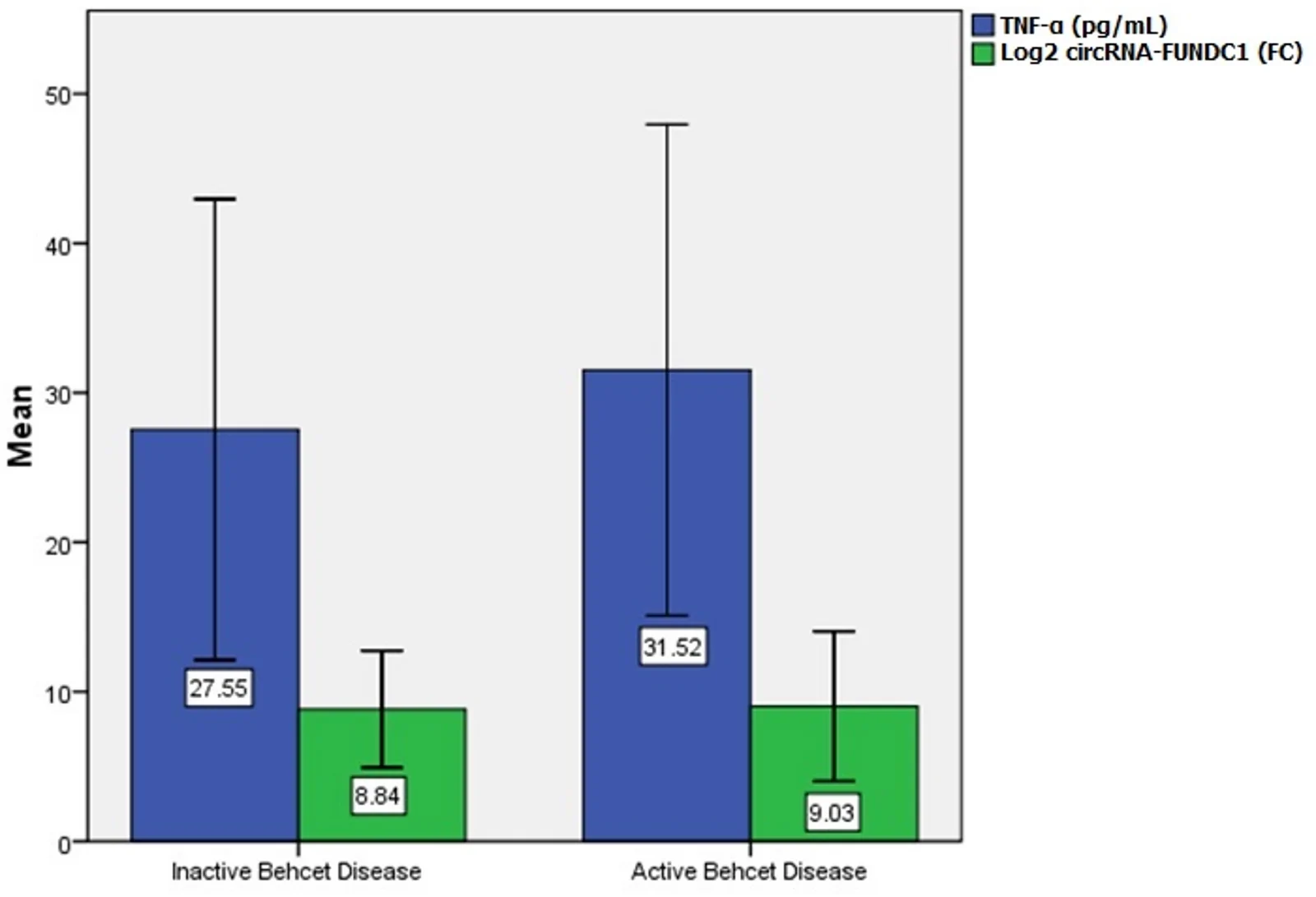
Association of circRNA-FUNDC1 and TNF-α with disease activity.

Based on the BDCAF Patient’s Index Score, patients were subclassified into inactive and active BD, and after evaluating the circRNA-FUNDC1 and TNF-α levels in serum accordingly, it was noticed that as disease activity gets worse, the levels of both biomarkers increase. For the expression of circRNA-FUNDC1 (FC) and the concentration level of TNF-α, tended to elevate in active BD groups than in active BD patients (Fig. 2).

### Correlations between the serum levels of circRNA-FUNDC1 and TNF-α with age and BDCAF Patient’s Index Score of patients with Behçet’s disease

Results showed that there were no correlations found between circRNA-FUNDC1 and the concentration level of TNF-α (*r*=−0.23, *p* = 0.11), circRNA-FUNDC1 and age (*r* = 0.03, *p* = 0.79), circRNA-FUNDC1 and BDCAF Patient’s Index Score (*r*=−0.07, *p* = 0.61), age and TNF-α (*r*=−0.09, *p* = 0.52), TNF-α and BDCAF score (*r* = 0.23, *p* = 0.09) among Behçet patients.

### circRNA-FUNDC1 and TNF-α: discriminatory performance between Behçet’s disease patients and healthy controls

**Fig. 3:**
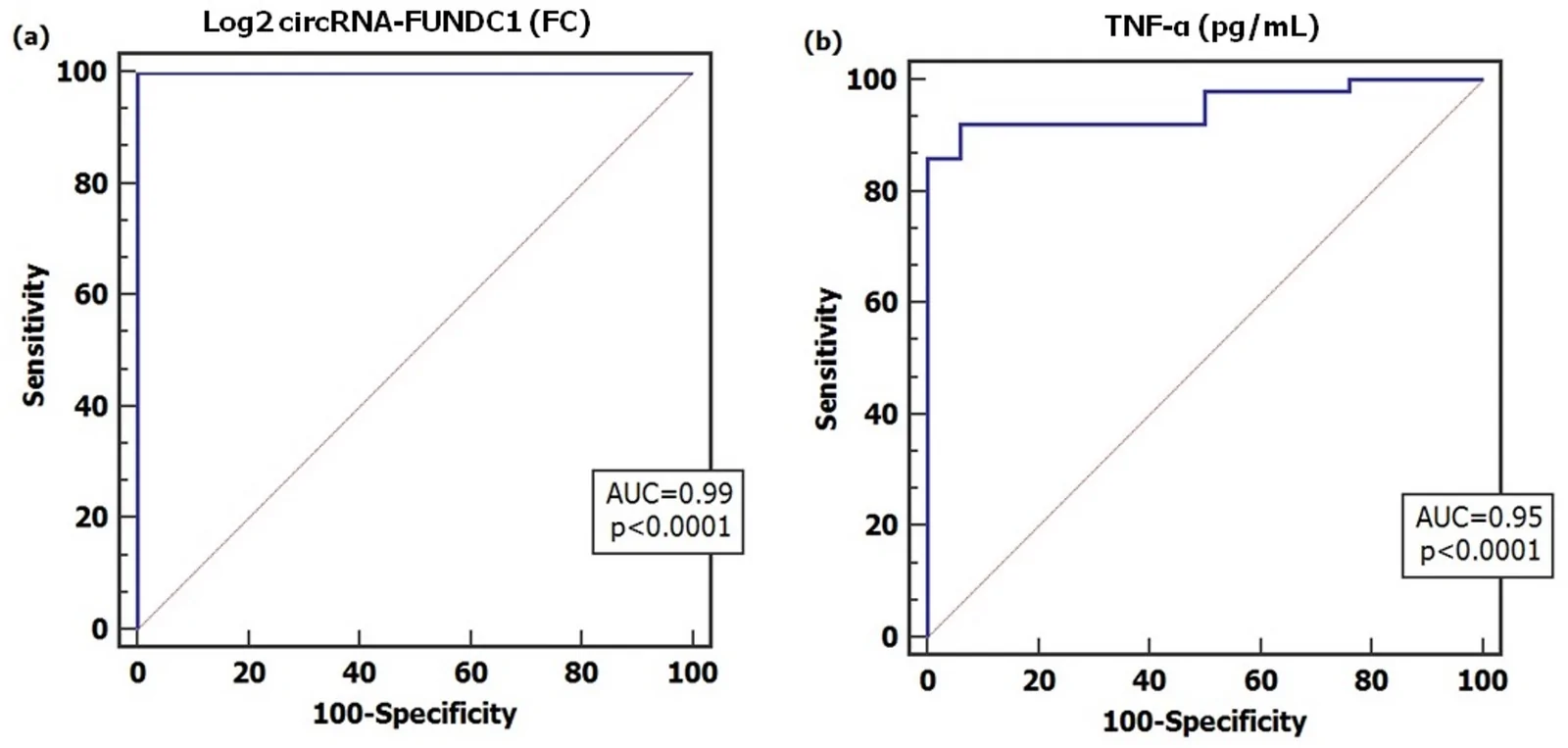
ROC curve analysis for (**a**) log2 circRNA-FUNDC1 (FC) and (**b**) serum concentration level of TNF-α.

The discriminatory performances of circRNA-FUNDC1 and TNF-α as biomarkers for Behçet’s disease were evaluated to discriminate patients from controls. The sensitivity for circRNA-FUNDC1 was 98% and the specificity was 100%, with a 99% accuracy (*p* < 0.0001) at a cutoff value of > 1.16 FC. For TNF-α, the sensitivity and specificity were 92% and 94%, respectively, with a 93% of accuracy at *p* < 0.0001 and a cutoff > 9.6 pg/mL. The receiver operating characteristic (ROC) curves of circRNA-FUNDC1 and TNF-α levels for serum of BD patients are shown in Table 4; Fig. 3.

**Table 4:** Discriminatory performance of circRNA-FUNDC1(FC) and TNF-α (pg/mL) between Behçet’s disease patients and healthy controls.

Biomarker	Log2 circRNA-FUNDC1(FC)	TNF-α (pg/mL)
AUC	0.99	0.95
Cut-off value	> 1.16 FC	> 9.6 pg/mL
Sensitivity	98%	92%
Specificity	100%	94%
Accuracy	99%	93%
95% CI a	0.96 to 1.0	0.88 to 0.98
p-value	< 0.0001*	< 0.0001*

^a^Binomial exact bold= significant (*P*≤0.05).

CircRNA-FUNDC1 showed excellent discriminatory performance in distinguishing Behçet’s disease patients from healthy controls in the present study and may represent a promising biomarker for Behçet’s disease. However, these findings should be interpreted with caution because disease control groups were not included. Further large-scale studies incorporating patients with other inflammatory and autoimmune diseases are required to validate the specificity and clinical utility of circRNA-FUNDC1 before it can be considered for routine diagnostic use.

In addition, regression analysis was used by which the models were intended to improve model stability in the presence of overlapping treatment regimens and unequal numbers of patients within treatment subgroups. Thus, medications that demonstrated significant associations in the univariate analyses where used in the models.

Hydroxychloroquine was independently associated with higher TNF-α levels (β = 17.20, *p* = 0.04). However, the model explained only 8.5% of the variability in TNF-α levels (Table 5).

**Table 5:** Medication-adjusted multivariate analysis for significant treatment variables.

(a) Model for TNF-α according to significant treatment variables						
Variable	β	95% CI lower	95% CI upper	*p*-value	*R*²	Adjusted *R*²
Constant	29.35	24.72	33.97	**< 0.001**	0.085	0.066
Hydroxychloroquine	17.20	0.84	33.55	0.04*		

(b) Model for circRNA-FUNDC1 according to significant treatment variables						
Variable	β	95% CI lower	95% CI upper	*p*-value	*R*²	Adjusted *R*²
Constant	6.28	3.31	9.25	**< 0.001**	0.179	0.125
Azathioprine	3.17	− 1.43	7.77	0.17		
Cyclosporin	− 0.32	− 5.07	4.42	0.89		
Steroid	2.02	− 2.28	6.32	0.35		

β: Regression coefficient, 95%CI: confidence intervals, R²: coefficients of determination. *Values are significant at *p* ≤ 0.05. Medication variables exhibited substantial overlap because many patients received combination therapy. Therefore, the regression coefficients should be interpreted cautiously.

On the other hand after adjustment for azathioprine, cyclosporin, and steroid use, none of the medications remained independently associated with circRNA-FUNDC1 expression (*p* > 0.05). The model explained 17.9% of the variability in circRNA-FUNDC1 levels (Table 5).

$$Regression={\text{ }}\beta 0+\beta 1X1+\beta 2X2+...\beta iXi$$

Where β0 is the constant intercept, X1 and X2 denote circFUNDC1 expression level of medication variable(s), and β1-βi are the estimated coefficients for each.

$$\begin{aligned} Regression\; equation\; for\; circRNA - FUNDC1 & =6.282+3.17\left( {Azathioprine} \right) \hfill \\ & \quad - 0.32\left( {Cyclosporin} \right)+{\text{ }}2.02\left( {Steroid} \right) \hfill \\ \end{aligned}$$

## Discussion

The Middle East, Mediterranean and the Far East regions had higher prevalence of Behçet’s disease, typically emerging in the third decade of life. As a chronic inflammatory disease, its exact cause remains unclear, but genetic predisposition, immune dysfunction, and environmental factors are key contributors^17–19^.

CircRNAs produced in human cells are key elements of the powerful molecular network regulating gene expression. CircRNAs are produced from many chromosomes, including chromosomes 1,2,3, and 6, where these chromosomes regulate immune and inflammatory pathways through mechanisms like microRNA sponging and gene modulation^6,20–22^.

No existing research, to our knowledge, has reported on circRNA-FUNDC1 in Behçet’s disease. However, given the established role of circRNAs in immune system regulation and inflammatory diseases, circRNA-FUNDC1 may contribute to Behçet’s disease pathogenesis^23^.

TNF-alpha contributes significantly to the inflammatory cascade of Behçet’s disease, driving immune system hyperactivity and tissue damage. It promotes neutrophil activation, increases vascular permeability, and enhances the release of other inflammatory mediators, contributing to BD’s hallmark symptoms, such as oral ulcers, uveitis, and vasculitis^24,25^.

In this study, we evaluated serum of circRNA-FUNDC1 expression levels and the concentration level of TNF-α of Behçet patients as well as healthy individuals. Results revealed that both biomarkers were upregulated in the Behçet’s patient group compared to controls.

Currently, there is limited direct research linking circRNA-FUNDC1 to BD. However, its host gene (FUNDC1) plays a pivotal function in mitophagy regulation, which is crucial for cellular homeostasis and has implications in various diseases. A review by Li et al. (2023) discusses the involvement of 31 different circRNAs in processes like autophagy, apoptosis, inflammation and oxidative stress following (IS) ischemic stroke. This highlights the potential of circRNAs as therapeutic targets and biomarkers in inflammatory conditions^7,26^.

In addition, circRNA-FUNDC1 has been previously studied in ischemic stroke, where it has been shown to regulate endothelial cell injury, indicating that knockdown of circRNA-FUNDC1 alleviates oxygen-glucose deprivation-induced damage in microvascular endothelial cells of the brain by targeting the microRNA-375/PTEN axis. This suggests that circRNA-FUNDC1 may contribute to inflammatory responses in ischemic stroke by modulating key molecular pathways^23,27^.

TNF-α is a well-established pro-inflammatory cytokine that plays a central role in the immunopathogenesis of Behçet’s disease (BD), contributing to immune dysregulation and persistent inflammation. Increased TNF-α production by CD4 + and CD8 + T cells, macrophages, and natural killer cells, together with elevated serum TNF-α levels in patients with BD compared with healthy controls, has been consistently reported, supporting its role in disease activity and maintenance^14,28,29^.

Similar elevations have also been observed in other autoimmune inflammatory diseases, such as juvenile chronic arthritis (JCA), further highlighting the broader pathogenic role of TNF-α in chronic inflammatory disorders^30^.

In addition, when measuring the serum biomarkers concerning the disease severity, it was detected that circRNA-FUNDC1 and TNF-α levels tended to elevate with the severity of the disease activity, where the BDCAF score gets higher in active behcet patients than inactive patients.

In agreement with our findings, a study involving 30 Egyptian Behçet’s patients and 20 healthy subjects, researchers found that TNF-α levels in serum were higher in patients, particularly those with active disease^28^.

Several studies have demonstrated that the BDCAF scores were higher in active patients when compared to inactive patients. A study involving 74 BD patients found that 16.2% were classified as active based on BDCAF scores, while 83.8% were inactive. BDCAF was higher in active patients than inactive individuals, concluding it to be a crucial factor contributing to functional disability in BD patients^31^.

Results also revealed a significant variation between circRNA-FUNDC1 and patients with negative musculoskeletal and CNS manifestations, with higher expression levels of circRNA-FUNDC1 than in positive patients. Although this finding appears counterintuitive, it may reflect the heterogeneous clinical nature of Behçet’s disease and the possibility that circRNA-FUNDC1 is differentially regulated among distinct disease phenotypes rather than being uniformly associated with all manifestations. Additionally, the relatively small number of patients with musculoskeletal and CNS involvement may have influenced these subgroup analyses. Another possible explanation is that circRNA-FUNDC1 may exert a compensatory or protective response in certain clinical settings. Therefore, these findings should be interpreted cautiously and warrant further investigation in larger, multicenter studies. In addition, the level of circRNA-FUNDC1 tended to be significantly higher in patients who received Azathioprine as an immunosuppressant than in those who did not. MeanWhile patients who did not take Cyclosporin had higher levels of circRNA-FUNDC1 than those with negative intake of Cyclosporin, with a slightly significant difference between groups. Our results also revealed that there was a significantly higher level of circRNA-FUNDC1 in patients who received Steroids than in those who did not.

A substantial proportion of patients received combination immunosuppressive therapy, and some medication categories contained relatively few patients in either the treatment or non-treatment groups resulting in unstable regression estimates and increase the likelihood of multicollinearity. Consequently, the independent effects of individual medications on biomarker expression could not be completely disentangled. Therefore, the findings from the medication-adjusted models should be interpreted cautiously and require confirmation in larger independent cohorts.

Our findings showed that for the concentration level of TNF-α, patients with oral ulcers had higher levels of TNF-α than those who did not, with a significant difference between both groups. As for organ manifestations, a significant difference was detected as regards patients with skin manifestations involvement that those who had negative skin issues. Also, TNF-α concentration levels tended to be higher in patients who received Hydroxychloroquine as an immunosuppressant.

A study analyzing 60 BD patients showed that those with active oral ulcers had significantly higher TNF-α levels compared to those without ulcers. These findings highlight TNF-α as a key inflammatory marker in BD, suggesting its potential role as a biomarker for the activity of the disease^32^.

Another study examined 45 BD patients reported significantly elevated TNF-α levels in the gingival crevicular fluid of those with recurrent oral ulcers. The results support TNF-α involvement in BD-related mucosal inflammation, reinforcing its potential as a therapeutic target^33^.

In agreement with our findings, a study involving 37 BD patients, of whom 19 had active oral ulcers, found that those with ulcers exhibited significantly higher salivary levels of TNF-α compared to patients without ulcers and healthy controls. This suggests that elevated salivary TNF-α is associated with oral ulcer activity in BD^34^.

The observed associations between circRNA-FUNDC1 expression and clinical features of Behçet’s disease suggest a relationship with overall disease activity rather than specific organ involvement. In the present study, higher circRNA-FUNDC1 levels were observed in patients with active disease as reflected by higher BDCAF scores, supporting its potential role as a marker of systemic inflammatory burden in Behçet’s disease^17,35^. However, the variability observed across different clinical manifestations, including musculoskeletal and CNS involvement, indicates that circRNA-FUNDC1 is unlikely to serve as an organ-specific biomarker and may instead reflect heterogeneous immune–inflammatory and mitochondrial stress responses across Behçet’s disease phenotypes. The association with immunosuppressive therapy likely reflects confounding by indication, as patients receiving treatment typically represent more severe disease. Overall, these findings suggest that circRNA-FUNDC1 may act as a biomarker of disease activity and systemic inflammation in Behçet’s disease rather than a determinant of specific clinical manifestations.

Several associations reached nominal statistical significance in the unadjusted analyses however, none remained significant after correction for multiple testing, suggesting that these findings should be interpreted cautiously and require confirmation in larger independent cohorts.

Although a direct regulatory relationship between circRNA-FUNDC1 and TNF-α has not yet been established, there is a biologically plausible indirect connection through FUNDC1-mediated mitophagy and inflammatory signaling. FUNDC1 is a key receptor involved in mitophagy and mitochondrial quality control. Impaired mitophagy results in the accumulation of damaged mitochondria, excessive production of reactive oxygen species (ROS), and the release of mitochondrial damage-associated molecular patterns, which subsequently activate inflammatory pathways and promote the production of pro-inflammatory cytokines, including TNF-α^36,37^.

Moreover, several studies have highlighted the role of FUNDC1 in inflammatory diseases and demonstrated that FUNDC1-mediated mitophagy participates in the regulation of immune and inflammatory responses^37,38^. Since TNF-α is a central cytokine in the pathogenesis of Behçet’s disease and a major therapeutic target, it was included in the present study as a representative inflammatory marker to explore whether circRNA-FUNDC1 expression is associated with the inflammatory status of Behçet’s disease.

However, no significant correlation was observed between circRNA-FUNDC1 and TNF-α levels in our cohort. This finding suggests that circRNA-FUNDC1 may reflect a pathogenic mechanism in Behçet’s disease that is independent of the classical TNF-α-mediated inflammatory pathway. Therefore, the analysis of TNF-α should be considered exploratory, and no direct mechanistic interaction or causal relationship can be inferred from the present cross-sectional study^37,38^.

Although demographic data (age and sex) and selected clinical information, including family history and associated diseases/comorbidities, were collected for the healthy control group, other clinical variables were not systematically collected or controlled for. Therefore, the findings observed for circRNA-FUNDC1 ROC curve should be interpreted with caution, and future studies should include more comprehensive characterization of healthy controls and appropriate disease control groups to further validate these results.Therefore, further studies involving larger, multicenter populations and external validation are warranted to confirm the diagnostic utility and generalizability of circRNA-FUNDC1 as a biomarker for Behçet’s disease.

## Conclusions

In conclusion, the upregulated levels of circRNA-FUNDC1 and TNF-α in serum may have a potential role understanding of the molecular mechanisms underlying BD.

## Data Availability

The data that support the findings of this study are not included in the manuscript for ethical reasons but are available upon reasonable request from the corresponding author, Olfat Shaker [email: olfat.shaker@kasralainy.edu.eg].
